# Use of Daptomycin for the Treatment of Methicillin-Resistant Coagulase-Negative Staphylococcal Ventriculitis

**DOI:** 10.1155/2012/593578

**Published:** 2012-05-09

**Authors:** Mohamed Erritouni, Nessrine Ktaich, James J. Rahal, Deborah Figueroa, Jaime Nieto, Carl Urban, Noriel Mariano, Frank Eisinger, Jonathan Abayev, David Nicolau, David Rubin, Murisiku Raifu, Sorana Segal-Maurer

**Affiliations:** ^1^Division of Infectious Diseases, Department of Medicine, New York Hospital Queens, Flushing, NY 11355, USA; ^2^Department of Pharmacy, New York Hospital Queens, Flushing, NY 11355, USA; ^3^Division of Neurosurgery, Department of Surgery, New York Hospital Queens, Flushing, NY 11355, USA; ^4^Department of Pharmacy, Hartford Hospital, Hartford, CT 06102, USA

## Abstract

Coagulase-negative staphylococci (CoNS) are the main pathogens causing hospital-acquired external-ventricular-drain- (EVD-) and lumbar-drain- (LD-) associated meningitis and ventriculitis. The treatment of these infections can be challenging and may require combination of intraventricular and intravenous administration of antibiotics. Limited animal data demonstrate rapid daptomycin bactericidal activity, adequate penetration in the setting of inflamed meninges, and extended half-life in the ventricles Steenbergen et al. (2009). There are limited clinical data using daptomycin intravenously and/or intraventricularly for the treatment of central nervous system infections (CNS) Elvy et al. (2008), Stucki et al. (2007), Lee et al. (2008) and Wallace et al. (2009). We report here our experience in the treatment of an EVD-related infection.

## 1. Case Report

A 52-year-old female with history of hypertension, smoking, alcohol dependence, and coronary vascular disease with recent myocardial infarction was admitted due to acute change in mental status. The patient had a spontaneous intraventricular hemorrhage, and an emergency bedside ventriculostomy was performed. Her hospital course was marked by persistent fevers in spite of multiple negative blood, cerebrospinal fluid (CSF), and urinary cultures. She received empiric levofloxacin intravenously 750 mg daily and vancomycin 1 gram intravenously every 12 hours until day 9 of hospitalization. Her neurologic function steadily improved, and the ventriculostomy was discontinued on day 11. However, her CSF cultures on that day yielded coagulase-negative staphylococcal (CoNS) species. Vancomycin 1 gram and rifampin 600 mg intravenously were administered every 12 hours. On day 15, computerized tomography (CT) of the head demonstrated increased intraventricular hemorrhage requiring reinsertion of an external ventriculostomy. Repeated CSF analysis demonstrated elevated WBC (57 cells/mm^3^ and protein 149 mg/dL) with decreased glucose (67 mg/dL). CSF cultures yielded CoNS again. Cultures of CSF continued to yield CoNS for the next three days despite the addition of daptomycin intravenously at 10 mg/kg (dosed at actual body weight) daily. As a result, intraventricular daptomycin was added on day 18, 10 mg daily for the first two days and then every other day. Culture of CSF became sterile on day 25 following 7 days of daptomycin intravenous and intraventricular therapy in addition to continued administration of intravenous vancomycin and rifampin. The second ventriculostomy was discontinued on day 38 (as was rifampin administration). Intravenous daptomycin and vancomycin were discontinued on day 55 after 37 days of dual treatment. Mental status progressively improved during this time. 

 Daptomycin peak and trough levels in the CSF were measured on day 29, and day 30, respectively (correlating to day 10 following the start of intravenous and day 11 following the start of intraventricular daptomycin). The peak CSF level (following intravenous and intraventricular administration) was 6.30 mcg/mL on day 29 and the trough CSF level was 1.39 mcg/mL on day 30. The serum trough level on day 30 was 20.15 mcg/mL, and the minimum inhibitory concentration (MIC) of CoNS to daptomycin was <1 mcg/mL.

The remaining hospital course was complicated with respiratory failure requiring tracheostomy and ventilation support, ventilator-associated pneumonia, sacral pressure ulcer, and *Clostridium difficile* colitis. The patient was successfully discharged on day 65 to a rehabilitation center.

## 2. Discussion

Data in animal models support rapid bactericidal activity of daptomycin. Early studies of MRSA ventriculitis in animals demonstrate daptomycin achieving greater bactericidal activity, more rapid killing kinetics, longer intraventricular half-life than vancomycin, minimal cell wall lysis, and significant reduction in host inflammatory reaction and cortical injury [1]. Several clinical reports describe successful use of daptomycin for the treatment of various CNS infections (e.g., meningitis, septic brain emboli, EVD-associated ventriculitis, etc.) caused by gram-positive bacteria (e.g., methicillin-resistant *Staphylococcus aureus*, *Enterococcus faecalis*, etc.) [2–5]. Doses of daptomycin used ranged from 6 to 12 mk/kg for intravenous administration and 5 to 10 mg every other day for intraventricular administration. Daptomycin was chosen either due to failure or adverse events of standard antibiotics early on in the course of the infection. Recent pharmacokinetic study of a single intravenous 10 mg/kg dose in patients with indwelling external CSF shunts meningitis/ventriculitis demonstrated CSF penetration of 11.5% (corrected for protein binding) [6].

In our patient, the addition of intravenous daptomycin was precipitated by persistent growth of CoNS in spite of treatment with vancomycin and rifampin. The decision to add intraventricular administration of daptomycin was made due to continued neurosurgical need for the ventriculostomy and potential limited CSF penetration of intravenous daptomycin. We found trough CSF levels of daptomycin to be 2-fold greater than the MIC for CoNS with peak levels well over 6-fold greater. These levels were obtained in the setting of both intravenous and intraventricular administration of daptomycin, and we cannot comment on the penetration and/or efficacy of intravenous daptomycin alone in this setting (or the need to add intraventricular daptomycin routinely). The impact of synergy between rifampin and daptomycin was not quantitated in our patient, and we do not believe continued use of vancomycin provided additional benefit in this case. Our patient's outcome was similarly successful as previous CNS infections treated with daptomycin.

We feel more clinical data are needed to delineate consistent daptomycin CSF penetration and role for intraventricular administration of daptomycin in CNS infections in conjunction with removal of involved prosthetic devices.
